# Augmented detection of septal defects using advanced optical coherence tomography network-processed phonocardiogram

**DOI:** 10.3389/fcvm.2022.1041082

**Published:** 2022-11-29

**Authors:** Po-Kai Huang, Ming-Chun Yang, Zi-Xuan Wang, Yu-Jung Huang, Wei-Chen Lin, Chung-Long Pan, Mei-Hui Guo

**Affiliations:** ^1^Department of Electronic Engineering, I-Shou University, Kaohsiung, Taiwan; ^2^Department of Pediatrics, E-Da Hospital, Kaohsiung, Taiwan; ^3^Department of Medicine, College of Medicine, I-Shou University, Kaohsiung, Taiwan; ^4^Department of Applied Mathematics, National Sun Yat-sen University, Kaohsiung, Taiwan; ^5^Department of Medical Research, E-DA Hospital, Kaohsiung, Taiwan

**Keywords:** artificial intelligence, atrial septal defect, bispectrum, deep learning, heart murmur, ventricular septal defect

## Abstract

**Background:**

Cardiac auscultation is a traditional method that is most frequently used for identifying congenital heart disease (CHD). Failure to diagnose CHD may occur in patients with faint murmurs or obesity. We aimed to develop an intelligent diagnostic method of detecting heart murmurs in patients with ventricular septal defects (VSDs) and atrial septal defects (ASDs).

**Materials and methods:**

Digital recordings of heart sounds and phonocardiograms of 184 participants were obtained. All participants underwent echocardiography by pediatric cardiologists to determine the type of CHD. The phonocardiogram data were classified as normal, ASD, or VSD. Then, the phonocardiogram signal was used to extract features to construct diagnostic models for disease classification using an advanced optical coherence tomography network (AOCT-NET). Cardiologists were asked to distinguish normal heart sounds from ASD/VSD murmurs after listening to the electronic sound recordings. Comparisons of the cardiologists’ assessment and AOCT-NET performance were performed.

**Results:**

Echocardiography results revealed 88 healthy participants, 50 with ASDs, and 46 with VSDs. The AOCT-NET had no advantage in detecting VSD compared with cardiologist assessment. However, AOCT-NET performance was better than that of cardiologists in detecting ASD (sensitivity, 76.4 vs. 27.8%, respectively; specificity, 90 vs. 98.5%, respectively).

**Conclusion:**

The proposed method has the potential to improve the ASD detection rate and could be an important screening tool for patients without symptoms.

## Introduction

Congenital heart disease (CHD) is one of the major causes of morbidity and death in children. Although CHDs may be associated with chromosomal or genetic anomalies (1), most are idiopathic, with unpredictable occurrence. The prenatal diagnosis of critical CHDs can reduce the risk of death attributable to cardiovascular compromise (2). Additionally, early detection of critical CHD can be achieved via pulse oximetry measurement within 48 h of birth (3). The Cochrane database review reported that pulse oximetry is a highly specific and moderately sensitive test for the detection of critical CHD (4). Nevertheless, only critical or cyanotic CHD can be detected by pulse oximetry measurements. Neither fetal ultrasonography nor neonatal pulse oximetry measurement can increase the detection rate of non-critical CHD. The detection of non-critical CHDs remains dependent on the physicians’ alertness before referral to cardiologists. However, non-critical CHDs can lead to congestive heart failure or sudden death later in life.

Delayed diagnosis of a non-critical CHD is not uncommon. Pfammatter and Stocker reported that of 323 patients, 32 had a delayed diagnosis of heart defects (5). The most common acyanotic CHDs with a delayed diagnosis were partial atrioventricular septal defects (3/10; 30%), followed by complete atrioventricular septal defects (6/35, 17%), coarctation of the aorta (5/32, 16%), and atrial septal defect (ASDs, 6/61; 10%) (6). Massin and Dessy investigated children with non-critical CHD and reported a delayed diagnosis in 8.7% of patients and in 35.1% of those requiring surgical or catheter-based interventions (7). We found delayed diagnosis in 27.5% of ASD and 4.4% of ventricular septal defect (VSD) cases. Furthermore, in many cases, the correct diagnosis was not made until adulthood, with some patients developing pulmonary hypertension before ASD was detected. These delays may preclude timely ASD repair. Therefore, early diagnosis provides practical benefits for the management of CHDs.

Septal defects may be detected by physical examination, electrocardiography, and/or chest radiography. Cardiac auscultation of heart sounds is the most common, simple, and effective method of detecting heart diseases with heart murmurs, and auscultation can be enhanced using an electronic stethoscope (8). However, the accuracy of interpretation remains dependent on the physician’s experience. Septal defects are often underdiagnosed, even when electrocardiograms and chest radiographs are used in combination as diagnostic tools (6). Additionally, although echocardiograms are readily available in many countries, they require cardiologists or professional technicians to perform the examination and analyze the outcomes. Therefore, a screening tool for septal defects that does not require cardiologist expertise is warranted. We aimed to construct an artificial intelligence-based interpretation model for the detection of septal defects in patients with heart murmurs.

## Materials and methods

### Participants

This prospective study was approved by the institutional review board of the E-DA Hospital. All participants and/or their guardians provided informed consent. The study was conducted at a tertiary medical center within a pediatric cardiology clinic. Participants had been previously diagnosed with heart diseases and underwent regular follow-up care or were referred by other physicians because of concerns regarding their conditions. Echocardiography was performed in all participants to confirm the diagnosis. Participants with patent foramen ovale or functional murmurs were excluded. Regarding ASDs and VSDs, the flow ratio between the pulmonary circulation and systemic circulation (Qp/Qs) was measured using echocardiography (9). For participants younger than 18 years, right ventricular dilation was defined by a z-score > 2.0 for the right ventricular end-diastolic area in the apical four-chamber view. Left ventricular dilation was defined by a z-score > 2.0 for the left ventricular end-diastolic area in the apical four-chamber view (10). For adults, right ventricular dilation was defined by a right ventricular end-diastolic volume (apical four-chamber view) normalized by body surface area > 87 mL/m^2^ for men and > 74 mL/m^2^ for women. Left ventricular dilation was defined by a left ventricular end-diastolic volume (apical four-chamber view) normalized by body surface area > 74 mL/m^2^ for men and > 61 mL/m^2^ for women (11).

This study included 216 participants. After excluding poor recordings (loud breathing sounds or faint heart sounds), 184 participants were analyzed, including 46 with VSDs, 50 with ASDs, and 88 with a normal heart structure. The median age was 8.58 years. The participants enrolled in the study were divided into the training dataset group and validation dataset group; there were 92 participants in each group and 552 recordings per group.

### Heart-sound recordings

**FIGURE 1:**
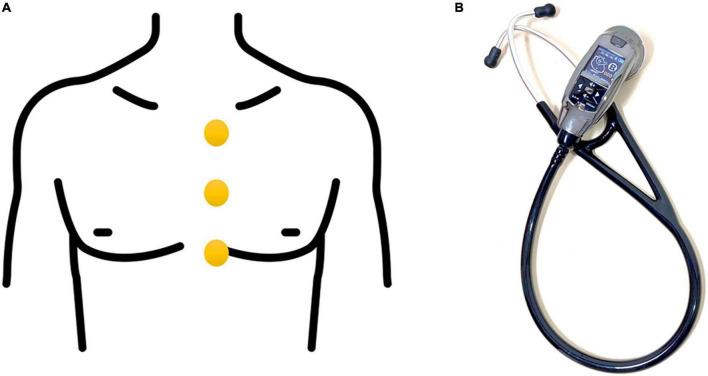
**(A)** The locations of the heart-sound recordings, including the left upper sternal border, left midsternal border, and left lower sternal border. **(B)** Electronic stethoscope used to collect heart-sounds.

Electronic heart-sound recordings were obtained by well-trained technicians using an electronic stethoscope (IMEDIPLUS DS101; B mode; frequency range, 20–200 Hz; sampling frequency, 8 kHz). Electronic heart sounds were recorded at the left upper sternal border (LUSB), left midsternal border (LMSB), and left lower sternal border (LLSB) (Figure 1). Heart sounds at each location were recorded twice, resulting in six phonocardiogram recordings for each participant. Each point was recorded for 10 s in the supine position in a standard examination room. Patients who were unable to cooperate during the recording of heart sounds were excluded.

Recordings of heart sounds from each participant in the training dataset group were used to construct the deep learning model. Heart sounds recordings from the participants in the validation dataset group were used to validate the accuracy, sensitivity, and specificity of the model. The electronic recordings of normal heart sounds (270 recordings) and ASD heart sounds (144 recordings) in the validation dataset were selected and allocated to the developed deep learning model and two pediatric cardiologists who independently listened to the heart sound recordings. Cardiologists were asked to distinguish normal heart sounds from ASD murmurs after listening to the electronic sound recordings. The cardiologists did not review the electrocardiograms or perform cardiac auscultation directly on patients. The interpretations of the two cardiologists were recorded as “normal” if there was agreement on normal. The interpretations were recorded as “ASD” if one of the cardiologists considered the heart sound as “ASD.” These results were used to calculate the detection sensitivity and specificity. The cardiologists also listened to 270 recordings of normal heart sounds and 138 recordings of VSD heart sounds in the validation dataset. Sounds were recorded as “VSD” or “normal,” and these recordings were also allocated to the deep learning model to identify them as normal or VSDs.

### Sound suppression

The electronic signals of heart sounds were denoised before the heart sounds were segmented to reduce the noise generated during the collection process. Throughout this study, we used the adaptive wavelet threshold for noise reduction in addition to performing segmentation.

The discrete wavelet transform comprises multi-resolution decomposition that separates information into different frequency bands (12). We decomposed the one-dimensional heartbeat signal using discrete wavelet conversion, reduced the noise, and used inverse one-dimensional wavelet conversion to restore the noise-induced heartbeat signal. One-dimensional discrete wavelet conversion was performed using filters, namely, a low-pass filter and a high-pass filter; the input signal was passed through these two filters and then sampled in halves to obtain the approximation coefficient and detailed coefficient (13).

### K-means clustering

**FIGURE 2:**
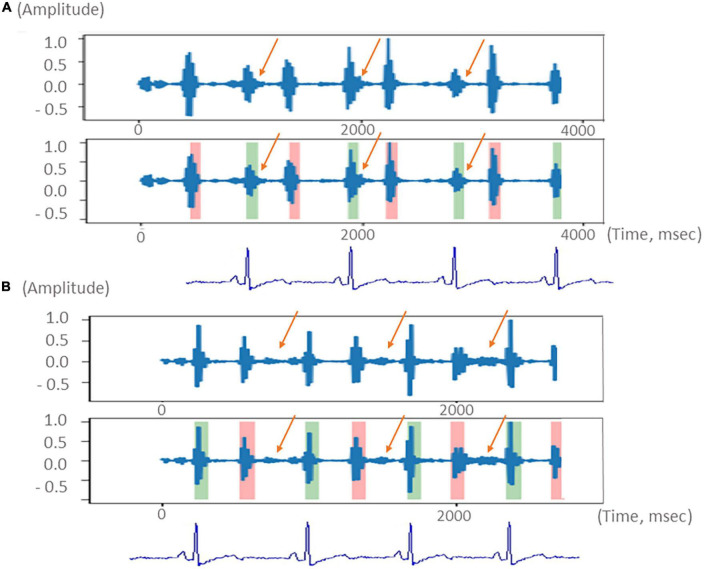
Schematic diagram of the segmentation of the heart sound from a patient with a systolic murmur (**A**, upper) and a patient with a diastolic murmur (**B**, lower). Green color indicates the heart sound S1. Pink color indicates the heart sound S2. The systolic and diastolic murmurs are indicated with orange arrows. Electrocardiogram used to demonstrate the systolic and diastolic phases is shown at the bottom.

K-means clustering is an unsupervised machine-learning algorithm that is frequently used to group similar data points together identifying underlying patterns. K-means + + is based on the K-means algorithm; however, it utilizes smart centroid initialization by randomly selecting random k-points during the first step of the process (14). This method was applied as a means of recognizing the systolic and diastolic phases during the cardiac cycle and to detect heart murmurs outside of normal heart sounds. Figure 2 shows schematic diagrams of the heart sound segmentation results of a patient with a systolic murmur (Figure 2A) and a patient with a diastolic murmur (Figure 2B) after noise reduction. The manipulation of the characteristic intervals of heart sounds is also shown.

### Bispectrum

**FIGURE 3:**
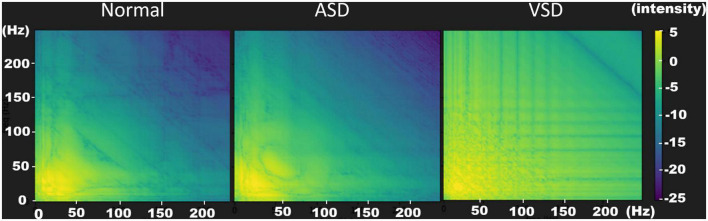
Bispectrum images of normal **(left)**, ASD **(middle)**, and VSD **(right)** sounds. The bispectrum images provide optimal resolutions to distinguish the three heart sound patterns. In a normal heart sound, only S1 and S2 contributes to the sound signal. Therefore, the color mapping of the bispectrum contains mostly low-intensity colors (dark green to purple). In ASDs and VSDs, the murmurs also contribute to the sound signals, which results in the interactions at the different frequency bands exhibiting a higher intensity in the bispectrum color mapping. There are more yellow and light green parts in the ASD bispectrum than in the normal bispectrum. These changes are more significant in the VSD bispectrum; therefore, many more yellow and light green parts are observed. ASD, atrial septal defect; VSD, ventricular septal defect.

To extract features, we used the bispectrum to convert the segmentation signal into a two-dimensional image associated with heart sound intervals (15). Using the Fourier transform, the waveform of a sound signal was decomposed into the intensities of its constituent frequency bands. This transform constituted the horizontal and vertical coordinates on the bispectrum. The interaction intensities between each frequency band (horizontal and vertical coordinates) were then transformed into color mappings of the bispectrum. As shown in Figure 3, yellow and light green colors represent high intensity, whereas purple and deep blue colors represent low intensity. In a normal heart sound, only S1 and S2 contributed to the sound signal, which simplified the interactions of the frequency bands. Therefore, the color mapping of the bispectrum was mostly low-intensity colors (dark green to purple). In ASD and VSD, the murmurs also contributed to the sound signals, which led to the exhibition of higher-intensity interactions at different frequency bands on the bispectrum color mapping. There were more yellow and light green parts of the ASD bispectrum than of the normal bispectrum (Figure 3). These changes were more significant on the VSD bispectrum, and many more yellow and light green parts were observed. These differences in bispectrum color mapping allowed us to distinguish among normal, ASD, and VSD.

These converted bispectrum signals were entered into the advanced optical coherence tomography network (AOCT-NET) and then subsequently used to train the AOCT-NET to construct the models for heart-sound classification (normal, ASD, VSD) (16, 17). Once the model was established, the normal heart-sound and ASD heart-sound recordings from the validation group were inputted into the AOCT-NET model to determine whether this could successfully distinguish normal heart sounds from ASD murmurs. Similarly, normal heart sounds and VSD heart sounds from the participants in the validation group were also entered into the AOCT-NET model to determine whether this could differentiate normal heart sounds from VSD murmurs.

### Statistical analyses

Statistical analyses were performed using SPSS Statistics version 22 (IBM SPSS Statistics for Windows version 22.0; IBM Corp., Armonk, NY). Two-tailed Student’s *t*-tests were used to compare the means of two independent datasets. The chi-square test was used to determine whether there were non-random associations between two categorical variables. Conversely, if the sample size was small (*n* < 5) in a 2 × 2 contingency table, the Fisher’s exact test was used to test the associations between two categorical variables. Sensitivity was defined as the number of defects detected by a screening method divided by the number of defects observed using echocardiography. Specificity was defined as the number of normal heart sounds determined using the screening method divided by the number of normal echocardiograms. The Kappa coefficient was used to test consistency between the screening method and echocardiography. *P* < 0.05 was considered to indicate statistical significance.

## Results

Among the 50 patients with ASDs, right ventricular dilation was observed in 32 (64%) patients, and none of the patients had tricuspid regurgitation that was more than moderate. Fourteen patients had pulmonary hypertension with a tricuspid regurgitation velocity greater than 2.7 m/s. Among patients with ASDs, 16 had an estimated Qp/Qs ratio < 1.5, and 34 had an estimated Qp/Qs ratio ≥ 1.5. Of the 46 cases of VSD, 26 were peri-membranous type, 11 were muscular type, and 9 were outlet type. Thirty-seven (80%) patients with VSDs had left ventricular dilation on echocardiography. Of these, 20 patients had an estimated Qp/Qs ratio < 1.5, and 26 patients had an estimated Qp/Qs ratio ≥ 1.5. Patient characteristics of the training and validation groups were similar (Table 1).

**TABLE 1:** Clinical characteristics of participants in the training and validation groups.

	Training group *n* = 92	Validation group *n* = 92	*P*-value
Male sex	53	49	0.78
Age (years), median	8.43	9.61	0.47**
1–5 years	9	14	0.27
6–10 years	41	34	0.35
11–15 years	32	35	0.44
16–55 years	10	9	0.87
Normal	43	45	0.94
ASD	26	24	0.94
Defect size-to-body weight ratio < 0.2	12	8	0.36
Defect size-to-body weight ratio 0.2–0.4	10	8	0.71
Defect size-to-body weight ratio > 0.4	4	8	0.19*
Right ventricle dilation	15	17	0.33
Qp/Qs ratio < 1.5	9	7	0.78
Qp/Qs ratio ≥ 1.5	17	17	0.99
VSD	23	23	0.94
Mild left ventricle dilation	12	13	0.77
Moderate left ventricle dilation	8	4	0.33*
Qp/Qs ratio < 1.5	9	11	0.76
Qp/Qs ratio ≥ 1.5	14	12	0.67

Pearson’s chi-square test, *Fisher’s exact test, and **Student’s t test were performed in the statistical analysis.

When evaluating the electronic recordings of heart sounds of the validation group, cardiologists recognized 44 ASD murmurs, including fixed S2 splitting in 21 cases. Among the 44 ASD murmurs identified, all were detected in patients with Qp/Qs ≥ 1.5. Furthermore, cardiologists detected 117 VSD murmurs and, of these, 73 murmurs were detected in patients with Qp/Qs ≥ 1.5 and 44 in patients with Qp/Qs < 1.5.

The AOCT-NET was capable of differentiating VSD murmurs from normal heart sounds as successfully as the assessment by cardiologists (Tables 2, 3). Additionally, 21 recordings considered normal by the cardiologists were identified as VSDs by echocardiography, while only 9 recordings were misinterpreted as normal by AOCT-NET (Table 2). The AOCT-NET demonstrated increased sensitivity in the detection of VSD murmurs compared to cardiologist assessment (Table 3). Overall, the accuracy rate of the cardiologist assessment was marginally greater than that using the AOCT-NET, although not significantly. Moreover, both methods had high consistency with the original echocardiographic findings, as shown by the Kappa coefficients outlined in Table 4.

**TABLE 2:** Results of VSD and ASD detection using the AOCT-NET and cardiologist assessment.

	Echocardiography	
	Normal (*n* = 270)	VSD (*n* = 138)
**AOCT-NET**		
Normal	252	9
VSD	18	129
**Cardiologist assessment**		
Normal	270	21
VSD	0	117

	**Normal (*n* = 270)**	**ASD (*n* = 144)**

**AOCT-NET**		
Normal	243	34
ASD	27	110
**Cardiologist assessment**		
Normal	266	104
ASD	4	40

	**Normal (*n* = 270)**	**ASD with RV dilation (*n* = 102)**

**AOCT-NET**		
Normal	243	12
ASD	27	90
**Cardiologist assessment**		
Normal	266	40
ASD	4	62

AOCT-NET, advanced optical coherence tomography network; ASD, atrial septal defect; RV, right ventricle; VSD, ventricular septal defect.

**TABLE 3:** The sensitivity, specificity, and accuracy of the AOCT-NET and cardiologists’ auscultation in detecting VSDs and ASDs.

	AOCT-NET	Cardiologist assessment
**VSD**		
Sensitivity	129/138 (93.5%)	117/138 (84.8%)
Specificity	252/270 (93.3%)	270/270 (100%)
Accuracy	381/408 (93.4%)	387/408 (94.9%)
**ASD**		
Sensitivity	110/144 (76.4%)	40/144 (27.8%)
Specificity	243/270 (90%)	266/270 (98.5%)
Accuracy	353/414 (85.3%)	306/414 (73.9%)
**ASD with RV dilation**		
Sensitivity	90/102 (88.2%)	62/102 (60.8%)
Specificity	243/270 (90%)	266/270 (98.5%)
Accuracy	333/372 (89.5%)	328/372 (88.2%)

ASD, atrial septal defect; RV, right ventricle; VSD, ventricular septal defect.

**TABLE 4:** The consistency of VSD and ASD detection between echocardiography and the screening methods.

	Echocardiography		Kappa coefficient	*P*-value
	Normal (*n* = 270)	VSD (*n* = 138)		
VSD by AOCT-NET	18	129	0.85	<0.001
VSD by cardiologist assessment	0	117	0.88	<0.001

	**Normal (*n* = 270)**	**ASD (*n* = 144)**		

ASD by AOCT-NET	27	110	0.67	<0.001
ASD by cardiologist assessment	4	40	0.33	<0.001

AOCT-NET, advanced optical coherence tomography network; ASD, atrial septal defect; VSD, ventricular septal defect.

AOCT-NET had greater screening sensitivity for detecting ASDs than cardiologist assessment (Tables 2, 3). Moreover, 104 recordings were interpreted as normal by the cardiologists, yet ASD was found on echocardiography, while only 34 ASD recordings were considered normal by AOCT-NET. Conversely, 27 normal heart sounds were misclassified as ASDs by AOCT-NET, while only 4 normal recordings were misclassified as ASDs by the cardiologists (Table 2). Furthermore, the Kappa coefficients outlined in Table 4 show greater consistency between AOCT-NET and echocardiography (0.67 vs. 0.33) than between cardiologist assessment and echocardiography. Therefore, as a screening tool, AOCT-NET can detect ASDs more accurately than cardiologists. Finally, among the 17 hemodynamically significant patients with ASDs with right ventricular dilation in the validation group (total recordings = 102), AOCT-NET had a sensitivity of 88.2% for detecting ASDs, while a 60.8% sensitivity was observed for cardiologist assessment (Table 3). Furthermore, 40 heart sounds were misdiagnosed as normal based on the assessment of the cardiologists.

## Discussion

This study developed a novel tool for detecting asymptomatic ASD patients that may be used in outpatient screening. This study determined that AOCT-NET had a higher sensitivity for detecting ASD murmurs than cardiologist assessment, although their screening sensitivities for detecting VSD murmurs were comparable.

The use of advanced diagnostic modalities, particularly echocardiography, has resulted in inadequate emphasis on cardiac auscultation (18). However, to date, cardiac auscultation remains the most cost-effective tool to successfully distinguish heart murmurs (19). Patients with VSD commonly experience holosystolic murmurs caused by turbulent blood flow between the left and right ventricles. Accurate detection of VSD by auscultation is generally easier than for ASD (19), as murmurs caused by ASD can often be much “quieter” depending on the defect size. However, auscultation is challenging, particularly with faint murmurs. The major aim of our study was to develop and explore a novel method to differentiate among normal, ASD and VSD murmurs using AOCT-NET. This method may allow early detection of both ASD and VSD, contributing to early treatment.

Automatic detection of valvular heart diseases based on higher-order spectral estimation and the bispectrum of phonocardiogram recordings is feasible. A 98% accuracy using this methodology has been reported for the diagnosis of heart valve diseases (17). Bispectrum color mapping has previously been applied to other medical fields, including electroencephalography recordings to enhance the prediction of seizures, and electrocardiogram recordings to characterize heart rate variability (20, 21). Bispectrum extract features can also be used to automatically differentiate normal heart sounds from heart sounds with pathological murmurs in newborns and children (22, 23). Wavelet transform, bispectrum, and optical coherence tomography were used in this study. Initially, the filtered signal was preprocessed to obtain the heart-sound segment for each patient, and the signals were converted into images through the bispectrum to detect non-linear frequency-band relationships between heart-sound signals. Later, we used images to train the AOCT-NET model, which led to its improvement. Accordingly, the model learned the frequency appearance of different types of CHD to achieve better classification results.

In this study, the AOCT-NET model distinguished ASD murmurs from normal heart sounds more accurately than cardiologist assessment, although the two methods displayed similar accuracy in detecting VSD (Table 3). This may be due to variations in the noises that are produced by the two types of defects and the ability of the cardiologist to detect these variations. With appropriate noise reduction, instruments such as digital stethoscopes can detect small differences in the amplitudes of sound waves, making them more sensitive than normal human hearing.

A recent study performed by Wang et al. using a novel algorithm constructed to identify systolic murmurs demonstrated high sensitivity (96%) and specificity (96.7%) for distinguishing VSD murmurs from normal heart sounds (24). A further study by Wang et al. successfully identified heart murmurs in children with CHD using artificial intelligence (25). However, these studies focused exclusively on detecting VSD. Our AOCT-NET model showed better performance in detecting ASD murmurs than the cardiologist assessment, with sensitivity increasing from 28 to 76% when the AOCT-NET was used.

When evaluating the sound recordings of patients with ASD in the validation group, cardiologists recognized a fixed S2 splitting in 21 sound recordings. Eighteen of the 21 electronic sound recordings were classified as ASDs by the AOCT-NET. Because the fixed S2 split also contributed to a sound signal, it also resulted in a change of the color mapping on the bispectrum. Although the color mapping change was subtle to human eyes, the AOCT-NET still recognized these sound recordings as ASDs. By combining the fixed S2 splitting and systolic murmurs reflected on the color mapping of the bispectrum, machine-learning has an advantage in detecting ASDs. In patients with ASD in the validation group with right ventricular dilation, 12 had mild or moderate tricuspid regurgitation, and 7 had pulmonary hypertension. The murmurs from tricuspid regurgitation and pulmonary hypertension increased the ability of both the AOCT-NET and the cardiologists to detect murmurs. AOCT-NET still had a higher sensitivity for patients with ASD with right ventricular dilatation than the cardiologist assessment (88.2 vs. 60.8%). The presence of right bundle branch block, defective T waves, and notch findings in the R wave of inferior derivations on electrocardiograms may also contribute to the detection of ASDs (26). Although auscultation combined with electrocardiograms reading may improve the detection rate of ASD, the low positive predictive value is a significant limitation, leading to lower specificity (27). Accordingly, early detection of ASDs remains a challenge in clinical practice.

Despite the capacity of the AOCT-NET to accurately identify VSDs and ASDs, technical difficulties were encountered as we developed the artificial intelligence-based heart murmur detection model. The original heart-sound recordings contained noises that had the potential to interfere with the characteristics of normal heart sounds and heart murmurs. To minimize the effect of these noises on our results, we utilized a Butterworth band-pass filter to produce the best output response. Automatic segmentation and classification of heart sounds were achieved with high accuracy and sensitivity using empirical wavelet transform-based phonocardiogram signal decomposition as previously described (28). Prior to denoising the phonocardiogram, the segmentation process facilitated the removal of irrelevant noise and emphasized the fundamental heart sounds (S1 and S2). An adaptive, non-linear mid-threshold estimation method for wavelet-based denoising of phonocardiograms has been proposed to efficiently suppress various types of noises (29, 30) and would be useful to implement in future studies. In addition to denoising, quick and accurate assessment of heart sounds can be achieved via wavelet transforms. To achieve this, the original heart sound signal is decomposed into a wavelet transform, and the wavelet decomposition coefficients of the signal are extracted (31). This classification method has been previously reported to successfully identify normal heart-sounds and heart murmurs (32).

The cost of developing the AOCT-NET was low because only an electronic stethoscope and a computer to transform the sound signal into optical tomography data were required. Machine-learning cannot replace echocardiography to confirm the final diagnosis of CHDs. However, machine-learning is a tool that physicians can use to improve the detection rate of heart murmurs, allowing referral of patients for further cardiac evaluation.

### Strengths and limitations

This study has some limitations. First, the sensitivity for detecting ASDs by AOCT-NET was limited because the tool could not recognize a cardiac lesion with no or slight heart murmurs, such as a patent foramen ovale or a small ASD. However, patients with minor heart diseases rarely need interventions. Moreover, screening with this method can reduce the number of undiagnosed cases of CHD, particularly for ASDs which are more difficult to recognize clinically. Further, patients with functional murmurs were excluded from this study, and cardiologist assessment was based on a dichotomous diagnosis, such as “normal or ASD” and “normal or VSD.” Nonetheless, the purpose of AOCT-NET was to enhance the screening sensitivity of non-critical CHDs. Furthermore, we achieved preliminary success in identifying heart murmurs. More studies are needed to differentiate functional murmurs from pathological murmurs. In the future, this model also has the potential to recognize other murmur types.

## Conclusion

The AOCT-NET can improve the detection of ASD murmurs and may be used as a screening tool for patients without symptoms and signs. This tool can be applied in clinical practice and contribute to the early detection and management of patients with ASDs.

## Data availability statement

The original contributions presented in this study are included in the article/supplementary material, further inquiries can be directed to the corresponding author.

## Ethics statement

The studies involving human participants were reviewed and approved by the institutional review board of the E-DA Hospital. Written informed consent to participate in this study was provided by the participants’ legal guardian/next of kin.

## Author contributions

P-KH, M-CY, Z-XW, W-CL, C-LP, and Y-JH performed the material preparation and data collection. P-KH, M-CY, Z-XW, and M-HG performed the analysis. P-KH wrote the first draft of the manuscript. All authors contributed to the study conception and design, commented on previous versions of the manuscript, and read and approved the final manuscript.
